# Prevalence of and factors associated with molar-incisor hypomineralisation in schoolchildren in the canton of Basel-Landschaft, Switzerland

**DOI:** 10.1007/s00784-022-04648-x

**Published:** 2022-07-26

**Authors:** Andreina Grieshaber, Tuomas Waltimo, Asin A. Haschemi, Judith Erb, Richard Steffen, Michael M. Bornstein, Eva M. Kulik

**Affiliations:** 1 Department of Oral Health & Medicine, University Center for Dental Medicine Basel UZB, University of Basel, 4058 Basel, Switzerland; 2 Department of General Pediatric and Adolescent Dentistry, University Center for Dental Medicine Basel UZB, University of Basel, 4058 Basel, Switzerland; 3 Department of Pediatric Oral Health and Orthodontics, University Center for Dental Medicine Basel UZB, University of Basel, 4058 Basel, Switzerland; 4 Private Practice, 8570 Weinfelden, Switzerland; 5 Department Research, University Center for Dental Medicine Basel UZB, University of Basel, Mattenstrasse 40, 4058 Basel, Switzerland

**Keywords:** Molar-incisor hypomineralisation, Children, Prevalence, Public oral health

## Abstract

**Objectives:**

As prevalence of molar-incisor hypomineralisation varies considerably in different countries and regions, the aim of this study was to obtain representative epidemiological data for schoolchildren living in the canton of Basel-Landschaft, Switzerland.

**Material and methods:**

A representative population of schoolchildren of three different age groups, i.e. 1st grade (mean age: 7.4 years), 6th grade (mean age: 12.6 years), and 9th grade (mean age: 15.7 years) visiting compulsory schools in the canton of Basel-Landschaft, Switzerland, was examined. The presence or absence of molar-incisor hypomineralisation at time of examination was recorded as well as potential influencing factors such as age group, gender, nationality, or the children’s place of residence.

**Results:**

A total of 1252 schoolchildren could be included. On average, the prevalence of MIH in the study population was 14.8%. No statistically significant differences were found for nationality, gender, or place of residence. Although not statistically significant, children from the youngest age group had the highest while children from the oldest age group had the lowest MIH prevalence.

**Conclusion:**

With a mean value of 14.8%, MIH prevalence among schoolchildren living in the canton of Basel-Landschaft, Switzerland, is comparable to mean values recorded globally.

**Clinical relevance:**

This study represents the first study on MIH prevalence in Switzerland and also provides further evidence on potential influencing factors.

## Introduction

Since the term molar-incisor hypomineralisation (MIH) was first introduced in the literature in 2001, this dental condition has increasingly become a relevant and important issue in dental medicine. Children suffering from this sometimes very painful enamel malformation present not only a challenge for dental professionals, but also for parents who are confronted with it on a daily basis [1–3].

MIH describes a qualitative enamel malformation in which discoloration and enamel fractures can occur due to the reduced mineralisation of inorganic components, i.e. calcium and phosphate. Originally, this condition was described for first molars and incisors only [4, 5], but recent studies show that all deciduous and permanent teeth can be affected [6]. The malformation of the first permanent molars and incisors takes place in a time frame from the 8th month of pregnancy to the 4th year of life, in accordance with the calcification of the respective tooth nuclei. This may explain the difficulty to pinpoint a specific cause for this disorder as symptoms only become apparent years after the damage has occurred. Although the exact aetiology is still not known, MIH appears to be multifactorial and acute or chronic illness and exposure to environmental pollutants during the last trimester of pregnancy and the first years of life have been suggested as potential causative and/or contributing factors [7–9]. Associated and predisposing factors are a major focus of research [10–12]. The investigation of potential treatment approaches has progressed significantly during the last two decades and current concepts are based on intensive prophylactic protocols as well as sealing or restoring the affected teeth [13–16].

As data collection is often based on different classification systems, study design, and age group analysed, comparison between epidemiological studies is difficult. Nevertheless, studies show that MIH is not a rare dental condition in children [17–20]. In a recent systematic review and meta-analysis, the mean global prevalence was estimated as high as 13.1% [19], while another systematic review reported a pooled prevalence of 14.2% globally [20]. Both studies noted significant differences between regions and countries, with particularly high MIH prevalence in some regions such as South America (18%) or Spain (21%) compared to Africa (11%) or India (8%). Regional differences can even be noticed within countries. In Germany, for example, prevalences between 4.3% and 14.7% have been recorded, and prevalence of first permanent molars was reported to be higher in children living in a city compared to children living in rural areas [21, 22].

Additionally, an increase in MIH prevalence is described in the literature. In a recent systematic review analysing results from 70 studies, children of younger age, i.e. < 10-year-olds, were shown to have a higher pooled prevalence than older children [20]. An increase in MIH prevalence affecting the first permanent molars was also observed in Germany comparing data from 2003 and 2015 [21].

Thus, factors associated with MIH are still debated among experts. Moreover, no data on the prevalence in Switzerland has been reported so far [23]. As prevalence varies highly in different countries and regions, the aim of this cross-sectional study was to obtain representative epidemiological data in schoolchildren living in the canton of Basel-Landschaft, Switzerland. Secondary objectives were to analyse the impact of various potential influencing factors such as age group or place of residence on MIH prevalence.

## Material and methods

### Study population and ethical approval

Based on regional law, surveys on the dental health of schoolchildren have to be performed regularly in the canton of Basel-Landschaft, Switzerland. Dental services for schoolchildren are organised by the cantons and are not standardised on a national level. A study protocol was submitted to the ethical committee Northwest and Central Switzerland (EKNZ 2021–00107), but the study was exempt from formal approval as the planned oral health check-ups were considered mandatory.

In the canton of Basel-Landschaft, compulsory education lasts 11 years. After 2 years at kindergarten, schoolchildren spend 6 years at a primary school before they switch for the last 3 years to a secondary school. Secondary school has three different sections where children are assigned based on their school performance: Section A (general requirements), Section E (extended requirements), and Section P (baccalaureate-level requirements).

As in the previous dental examinations in the canton of Basel-Landschaft, a random sampling of school classes from first, sixth, and ninth grades was performed [24]. This resulted in a study population with 1st grade schoolchildren who were in their first year of primary school, 6th grade schoolchildren attending the last year of primary school and 9th grade schoolchildren who were in their last year of compulsory schooling (Sections A, E, or P).

Prior to the dental examination, the legal guardians of the selected schoolchildren received information about the purpose and procedures of the examinations as well as a questionnaire to collect information on the date of birth, nationality, gender, and place of residence to complete the basic demographic information obtained by the Cantonal Statistical Office Basel-Landschaft.

### Dental examinations

Dental examinations were conducted by three teams consisting of one dentist and one dental assistant each. The setup of the dental examinations was in accordance with the principles of clinical oral health surveys as recommended by the World Health Organization [25]. That implies all examinations were performed in schoolrooms under artificial light (high-performance LED headlamps). The examination was announced in advance, so children would brush their teeth before class. If necessary, excess saliva was removed with a cotton swab by the dentist prior to examination which included a sickle probe (Maillefer Nr. 6, Maillefer, Ballaigues, Switzerland) and a plane mouth mirror.

MIH diagnosis was based on the evaluation system described by Steffen et al. [26]. Clinical manifestations ranging from Index 1 (MIH without hypersensitivity, without defect) to Index 4 (MIH with hypersensitivity, with defect) were recorded as MIH (yes / no), regardless of the affected tooth and the severity of the disease [26].

### Reliability

Prior to the start of the study, all examiners were trained and calibrated by a paediatric dentist (R.S.) specialised in the diagnosis and treatment of MIH. The reliability of the clinical examinations between pairs of examiners was assessed by comparing schoolchildren from two school classes (1st and 6th grade, respectively). Cohen’s kappa values for inter-rater reliability values were ranging from 0.72 to 0.89 for the three examiners. To assess the intra-rater reliability, 4.5% of the children were examined twice by the dentists. Cohen’s kappa values for intra-rater reliability were 1.0 for all three examiners, respectively.

### Data collection

All data were entered on-site in a Microsoft Access-based data entry programme (Microsoft Corporation, Redmond, WA, USA) including the information from the questionnaire.

Children were included in the evaluation only if all four first permanent molars were fully erupted. MIH values per child were presented as a dichotomous variable: 1 representing the presence of MIH (yes), and 0 representing the absence of MIH (no).

Age was calculated as the difference between the date of the dental examination and the date of birth.

The classification of the children’s places of residence into rural, intermediate or urban regions was based on the regional typology published by the Federal Statistical Office [27]. This classification uses a reproducible algorithm incorporating both morphological and functional criteria, such as density of inhabitants, employed persons or overnight stays and is comparable to similar approaches for other European or international countries. For Switzerland, relevant factors for this classification are the permanent residual population, jobs/employed persons and overnight stay equivalent in hotels. Additionally, an accessibility index is included for rural municipalities which is based on an algorithm that assesses the effort required to reach destinations outside the respective municipality. The exact method for classification is based on a nine-step procedure that is described in more detail in the publications of the Federal Statistical Office [28, 29].

Children with an incomplete dataset were excluded from the study. Prior to the statistical analysis, all data were anonymised.

### Statistical analysis

To identify possible influencing factors for MIH such as age, gender, nationality, place of residence, and the school level at 9th grade, individual logistic regressions were performed to estimate the odds ratio (OR) and the corresponding 95% confidence interval (CI). All of the tests performed were two-tailed tests with a 0.05 significance level.

The analyses were done using IBM SPSS Statistics for Windows Version 27 (IBM Corp. Armonk, NY, USA).

## Results

In total, 1822 children were asked to participate in this study. However, 465 schoolchildren (25.5%) were excluded as they were absent from class on the day of the dental examination, and a further 105 (5.8%) were excluded because not all four first permanent molars were present in their dentition. As only the basic demographic information obtained by the Cantonal Statistical Office was used for the present study, a total of 1252 children (68.7%) could therefore be included in this study. This corresponds to approximately 15% of all schoolchildren in the canton of Basel-Landschaft in these three age categories. The detailed characteristics of the study population are shown in Table 1.

**Table 1:** Descriptive characteristics of study participants. Shown are sample size, age, gender, nationality, and the respective regional typology for all participants and the three age categories

	N	Age (y)	Gender		Nationality		Regional typology		
		(min–max)	Male	Female	Swiss	Foreign	Urban	Intermediate	Rural
All children	1252	11.8 (6.5–17.9)	647 (51.7%)	605 (48.3%)	935 (74.7%)	317 (25.3%)	818 (65.3%)	266 (21.3%)	168 (13.4%)
1st grade	409	7.4 (6.5–8.6)	199 (48.7%)	210 (51.3%)	317 (77.5%)	92 (22.5%)	299 (73.1%)	58 (14.2%)	52 (12.7%)
6th grade	469	12.6 (11.6–14.0)	268 (57.1%)	201 (42.9%)	356 (75.9%)	113 (24.1%)	271 (57.8%)	122 (26.0%)	76 (16.2%)
9th grade	374	15.7 (14.4–17.9)	180 (48.1%)	194 (51.9%)	262 (70.1%)	112 (29.9%)	248 (66.3%)	86 (23.0%)	40 (10.7%)

About three-quarters of the children examined were of Swiss nationality, and the majority lived in an urban setting. This distribution was found to be similar for all three different age categories.

On average, the prevalence of MIH in the study population was 14.8%, with the highest prevalence in the youngest age category (Table 2). However, differences between age groups were not statistically significant as well as other potential influencing factors such as gender, nationality, or place of residence.

**Table 2:** MIH prevalence rates among age groups, gender, nationality, regions, and section at secondary school at 9th grade. Shown are the percentages and total numbers (in brackets) for children with MIH in the respective subgroup. Level at 9th grade indicates the respective section at secondary school in 9th grade with A = general requirements, E = extended requirements, and P = baccalaureate-level requirements. Risk comparisons are given as odds ratios (OR) with the respective 95% confidence intervals (CI 95%)

	MIH	OR (95% CI); *p* value
All children (*n* = 1252)	14.8% (*n* = 185)	
Age group		*p* = n.s.
1st grade (*n* = 409)	15.7% (*n* = 64)	1
6th grade (*n* = 469)	14.7% (*n* = 69)	0.93 (0.64, 1.35); *p* = n.s
9th grade (*n* = 374)	13.9% (*n* = 52)	0.87 (0.59, 1.29); *p* = n.s
Gender		*p* = n.s
Male (*n* = 647)	14.1% (*n* = 91)	1
Female (*n* = 605)	15.5% (*n* = 94)	1.12 (0.82, 1.57); *p* = n.s
Nationality		*p* = n.s
Swiss (*n* = 935)	14.5% (*n* = 136)	1
Foreign (*n* = 317)	15.5% (*n* = 49)	1.07 (0.75, 1.53); *p* = n.s
Regional typology		*p* = n.s
Urban (*n* = 818)	15.8% (*n* = 129)	1
Intermediate (*n* = 266)	13.9% (*n* = 37)	0.86 (0.58, 1.28); *p* = n.s
Rural (*n* = 168)	11.3% (*n* = 19)	0.68 (0.41, 1.14); *p* = n.s
Level at 9th grade		*p* = 0.04
level A (*n* = 100)	15.0% (*n* = 15)	1
level E (*n* = 142)	8.5% (*n* = 12)	0.52 (0.23, 1.17); *p* = n.s
level P (*n* = 132)	18.9% (*n* = 25)	1.32 (0.66, 2.67); *p* = n.s
level A (*n* = 100)	15.0% (*n* = 15)	1.91 (0.85, 4.28); *p* = n.s
level E (*n* = 142)	8.5% (*n* = 12)	1
level P (*n* = 132)	18.9% (*n* = 25)	2.53 (1.22, 5.28); *p* = n.s
level A (*n* = 100)	15.0% (*n* = 15)	0.76 (0.38, 1.52); *p* = n.s
level E (*n* = 142)	8.5% (*n* = 12)	0.40 (0.19, 0.82); *p* = 0.01
level P (*n* = 132)	18.9% (*n* = 25)	1

*n.s.* not significant

Logistic regressions showed that the level at 9th grade might be an influencing factor for MIH as a statistically significant difference could be noticed (*p* = 0.04). Therefore, the three subgroups were analysed in more detail. A statistically significant difference between level E and level P was present, with a lower MIH prevalence in children who attended a secondary school with an extended requirement profile (Section E) compared to children attending a school with baccalaureate-level requirements (*p* = 0.01).

## Discussion

This study represents the first study on MIH prevalence in Switzerland, and one of the few investigations on a large sample to assess potential influencing factors of the disease. With a mean value of 14.8%, MIH prevalence in schoolchildren living in the canton of Basel-Landschaft, Switzerland, is comparable to the global mean of 14.2% obtained by systematically analysing 70 studies [20] or to the mean prevalence of 13.1% reported by a similar systematic review that included 99 studies from 43 countries [19].

However, significant differences in the prevalence of MIH exist between continents, regions and even within countries. Geographical differences on different continents have been described by subgroup analysis with South America having the highest prevalence of MIH (18.0%), followed by Oceania (16.3%), Europe (14.3%), Asia (13.0%), and Africa (10.9%) [20]. Within Europe, mean values between 3.6% in Bulgaria and 37.3% in Denmark have been reported [30, 31]. Part of these differences might be explained by different study designs, age groups selected or criteria used to diagnose MIH. Nevertheless, even when controlling for these factors, regional differences still do exist. In a recent study conducted in Germany involving a total of 2395 children aged 7–10 years from four different cities, an overall prevalence of 10.1% was recorded. In this investigation, that included the same diagnostic guidelines for all calibrated examiners and a homogenous age group, statistically significant differences between the four selected German cities were noted, with MIH prevalence ranging from 4.3% to 14.6% [22]. The MIH prevalence for the canton of Basel-Landschaft obtained in this study might, therefore, not be representative for the whole of Switzerland [26].

Regional variations in MIH prevalence based on rural–urban comparisons have been described recently in schoolchildren living in different areas of Germany. The number of children with affected first permanent molars was reported to be higher in children living in a city (17.4%) compared to children living in rural areas (9.4%) [21]. Although not statistically significant, the same tendency was also noticed in the present study. Schoolchildren with an urban place of residence had a MIH prevalence of 15.8% compared to 11.3% for children living in a rural area.

It has been argued that such regional differences are due to genetic factors contributing to the MIH phenotype that may vary depending on geographic origin [11]. As prevalence varies even between neighbouring regions, as is also the case in the current study, this might suggest an even more complex interaction between multiple causes and risk factors. Accordingly, no definite risk factors have been identified so far and both genetic as well as environmental factors have been implicated to play a role in the development of MIH. A number of pre- and perinatal factors such as maternal smoking, medication use during pregnancy, prematurity, or birth complications have been investigated in detail; yet, for these factors, no conclusive association with the condition has been found. In contrast, most studies agree that early childhood illnesses, in particular febrile illnesses, most likely correlates with the occurrence of MIH [9].

Recently, the focus of research has shifted to the impact of DNA methylation, enamel forming and immune response-related genes. In this context, the interaction of two genes thought to contribute to oral clefts have been analysed. The interferon regulatory factor-6 (*IRF6*) is a transcription factor that has been shown to influence amelogenesis, while transforming growth factor alpha (*TGFA*) is an essential cell regulator and cytokine. Both gene–gene as well as gene-environment interactions were noticed. Additionally, associations differed in the cohorts analysed, supporting the hypothesis that genetic factors may contribute to MIH depending on geographic locations [11]. In the cord blood of twin pairs, differently methylated regions were identified in several genes that were associated with hypomineralised second primary molars at age of 6 years. Genes identified by these studies have previously been linked with dental tissue development [32]. Another focus of research lies on a localised exposure of immature enamel to serum albumin. It has been shown that, due to the binding of albumin, the growth of enamel-mineral crystals is blocked leading to the typical demarcated hypomineralisations [33, 34].

The prevalence as well as the severity of MIH appears to be increasing [21, 35]. In a longitudinal study, 182 children previously diagnosed with MIH were re-examined after 18 months. An increase of MIH-affected teeth was noticed in this cohort, and especially, teeth previously diagnosed with yellow and brown enamel opacities were at high risk for an increase in severity [35]. In another study, the prevalence of hypomineralised first permanent molars in children aged 6–12 years has been shown to increase from 5.9% in the school year 2002/2003 to 9.4% in the school year 2014/2015 in rural areas in Germany [21]. A potential increase in MIH prevalence could also be noticed in the present study, with children from the youngest age group (i.e. schoolchildren from 1st grade) having the highest while children from the oldest age group (i.e. schoolchildren from 9th grade) having the lowest prevalence. A repeated assessment of schoolchildren will allow to analyse the trend of MIH prevalence in the canton of Basel-Landschaft.

As recommended by Elfrink et al. (2015), the optimal examination for children included in MIH studies seems to be at 8 years. In the present study, schoolchildren from 1st grade had a mean age of 7.4 years which is consistent with this recommendation. Examining more age groups allows for cross-sectional comparison between different age groups in this population. However, in older age groups the prevalence of MIH might be underestimated as hypomineralisations might be masked by other defects such as dental caries [21]. Therefore, to confirm a potential increase in prevalence, repeated dental examinations in the same region over a longer time span using the same classification criteria in a similar age group and, ideally, also by the same examiners are needed.

Other factors analysed in the present study, such as gender or nationality, had no statistically significant effect on MIH prevalence. This is in agreement with a recent meta-analysis, where no statistical differences were found between males and females [20]. However, a statistically significant difference was noticed for the level at 9th grade. Schoolchildren attending Section E had a significantly lower MIH prevalence compared to those attending one of the two sections, namely Section A or Section P. However, only between 100 and 132 schoolchildren were included in these three different subgroups which is below the minimum of 300 children recommended for MIH prevalence studies. Therefore, this result could have been obtained by chance, and needs to be interpreted with some caution [17]. However, further analysis of a potential influence of the children’s social background and MIH prevalence including a larger number of children in the different subgroups might be of interest.

Another limitation of our study is that only the presence or absence of MIH was recorded, regardless of the affected tooth and/or the severity of the disease. For this reason, it is not possible to estimate the overall treatment need or potential differences in treatment needs between the three age groups included. This is also due to the ongoing corona pandemic, which has led to some restrictions in the clinical evaluation process. Therefore, further studies are needed to clarify these points and, in principle, the MIH treatment need index (MIH TNI) might be applicable to answer these questions as well as indicate if there is an increase in MIH prevalence [26].

## Conclusion

In summary, this study represents the first study on MIH prevalence in Switzerland. With a mean value of 14.8%, the MIH prevalence among schoolchildren living in the canton of Basel-Landschaft, Switzerland, is similar to mean values recorded globally. Children from the youngest age group had the highest while children from the oldest age group had the lowest MIH prevalence (15.7% vs 13.9%). Likewise, children living in urban areas had a higher mean MIH prevalence compared to children from rural areas (15.8% vs 11.3%).
